# Glycyrrhizic Acid Reduces Heart Rate and Blood Pressure by a Dual Mechanism

**DOI:** 10.3390/molecules21101291

**Published:** 2016-09-27

**Authors:** Kailash Singh, Aung Moe Zaw, Revathi Sekar, Ahuja Palak, Ahmed A. Allam, Jamaan Ajarem, Billy K. C. Chow

**Affiliations:** 1 School of Biological Sciences, The University of Hong Kong, Pokfulam Road, Hong Kong SAR, China; kailash@connect.hku.hk (K.S.); moezaw@connect.hku.hk (A.M.Z.); revathis@hku.hk (R.S.); palak@hku.hk (A.P.); 2 Department of Zoology, College of Science, King Saud University, Riyadh 11451, Saudi Arabia; aallam@ksu.edu.sa (A.A.A.); jajarem@KSU.EDU.SA (J.A.); 3 Department of Zoology, Faculty of Science, Beni-Suef University, Beni-Suef 62511, Egypt

**Keywords:** adrenergic receptors, glycyrrhizic acid, vasoactive-intestinal peptide, tachycardia

## Abstract

Beta adrenergic receptors are crucial for their role in rhythmic contraction of heart along with their role in the pathological conditions such as tachycardia and high risk of heart failure. Studies report that the levels of beta-1 adrenergic receptor tend to decrease by 50%, whereas, the levels of beta-2 adrenergic receptor remains constant during the risk of heart failure. Beta blockers—the antagonistic molecules for beta-adrenergic receptors, function by slowing the heart rate, which thereby allows the left ventricle to fill completely during tachycardia incidents and hence helps in blood pumping capacity of heart and reducing the risk of heart failure. In the present study, we investigate the potential of glycyrrhizic acid (GA) as a possible principal drug molecule for cardiac arrhythmias owing to its ability to induce reduction in the heart rate and blood pressure. We use in vitro and in silico approach to study GA′s effect on beta adrenergic receptor along with an in vivo study to examine its effect on heart rate and blood pressure. Additionally, we explore GA′s proficiency in eliciting an increase in the plasma levels of vasoactive intestinal peptide, which by dilating the blood vessel consequently, can be a crucial aid during the occurrence of a potential heart attack. Therefore, we propose GA as a potential principal drug molecule via its potential in modulating heart rate and blood pressure.

## 1. Introduction

β-Adrenergic receptors (β-AR) which belong to the superfamily of membrane receptors called G-protein coupled receptors (GPCRs) are known to interact with G-protein in order to activate the downstream signaling processes [1]. β-ARs are well known to have a widespread influence on cardiac functions [2], modulating both contraction and relaxation [3] upon its interaction with epinephrine and norepinephrine, the primary agonists for all the β-ARs [4]. Therefore, the use of β-adrenergic agonists/antagonists has displayed a great potential in clinical practice [5] wherein, β-AR antagonists or beta blockers are well recognized to slow down the heart rate by blocking the effect of the hormone adrenaline [6].

There exist four subtypes of β-AR—β1-adrenergic receptor (β1-AR), β2-adrenergic receptors (β2-AR), β3-adrenergic receptors (β3-AR) and β4-adrenergic receptors (β4-AR) [4]. β1-AR, β2-AR and β3-AR are known to be expressed in heart, with 70%–80% of all β-AR being β1-AR [7]. Also, β2-ARs constitute 20%–25% of the total β-AR expression. On the contrary, β3-AR is known to be primarily expressed in adipose tissue and minimally in heart. β4-AR, however, is acknowledged to be a low affinity state of β1-AR, with more studies and characterization yet to be done [8]. The functional analysis of these different subtypes has been elucidated using knockout models, wherein, β1-AR knockout and the β2-AR knockout mice models have shown typical responses upon catecholamine stimulation [9]. Additionally, the knock out mice models are reported to have no developmental defects when compared to the wild type [10]. Interestingly, the signaling changes observed during progression of heart failure majorly involve reduction of up to 50% of β1-AR; β2-AR levels however, remain constant [1,11]. Furthermore, β2-AR is reported to mediate the vasodilator response during hypertension [12,13,14]. Conclusively, recent evidence depict that β2-AR is particularly significant in a number of pathological conditions [15,16,17]. For instance, β1-AR sensitivity substantially drops during chronic heart failure, whereas, the levels of β2-AR remains constant, making the failing heart more dependent on β2-AR for inotropic support [15,16]. Therefore, β2-AR antagonist shall be of great use for the prevention of the reoccurrence of a heart attack [18].We have validated GA, a plant extract, to be a potent antagonistic molecule in vitro, which upon administration in mice model leads to a drop in the heart rate and blood pressure peaking after 30 min of administration. Likewise, we observed elevated levels of vasoactive intestinal peptide (VIP) after 30 min of GA administration. GA, a widely studied compound, is extracted from the root extracts of *Glycyrrhiza glabra* (liquorice) [19]. It is well known for many of its medicinal functions, and is often associated with ulcers [20], inflammation [20,21,22], bronchitis [23], diabetes [24,25], cancer [26,27] and hepatitis [28,29,30]. However, it is known to have a long term hypertensive effect in humans and vast amount of toxicological studies have been conducted previously which strongly suggests GA to be chronically precarious [31]. Nevertheless, there is no known acute toxicological implication of GA. Also according to Hazardous Substances Data Bank (http://toxnet.nlm.nih.gov/cgi-bin/sis/search2/r?dbs+hsdb:@term+@DOCNO+496), the GA related toxicity “only develops after chronic excessive ingestion. No specific monitoring is needed after single acute overdose”.

## 2. Results

### 2.1. In Vitro cAMP Assay

The effect of 10 µM GA on the cAMP levels of CHO cells transfected with β-ARs (β1-AR, β2-AR and β3-AR) and stimulated by Nor-epinephrine (0.1 µM) was tested.

**Figure 1:**
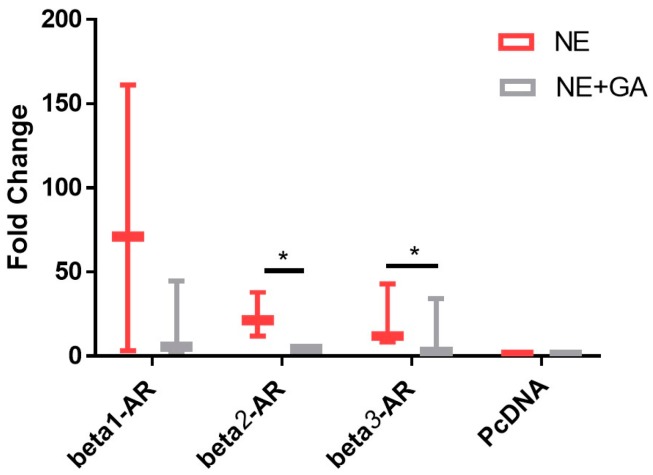
Fold change in the cAMP levels upon activation of adrenergic receptors by 0.1 μM Nor-epinephrine alone (NE) alone and activation of adrenergic receptors by 0.1 μM Nor-epinephrine in the presence of 10 µM (GA) glycyrrhizic acid (NE + GA). GA along with NE results in a statistically significant reduction in the cAMP levels when compared with NE alone in CHO overexpressed with Beta 2-adrenergic receptor (*p* value = 0.041) and Beta 3-adrenergic receptor (*p* value = 0.021) (N = 3, *n* = 9). * *p* < 0.05.

We did not observe any statistically significant reduction in the cAMP levels in β1-AR. However, a statistically significant drop in the cAMP levels was observed in cells transfected with β2-AR and β3-AR and subsequently stimulated with nor-epinephrine (NE), when compared to β2-AR and β3-AR transfected cells and stimulated with nor-epinephrine (NE) along with 10 µM of GA, suggesting selective antagonistic capability of GA against β2-AR and β3-AR (Figure 1).

### 2.2. Acute Effect of GA on Heart Rate & Blood Pressure

**Figure 2:**
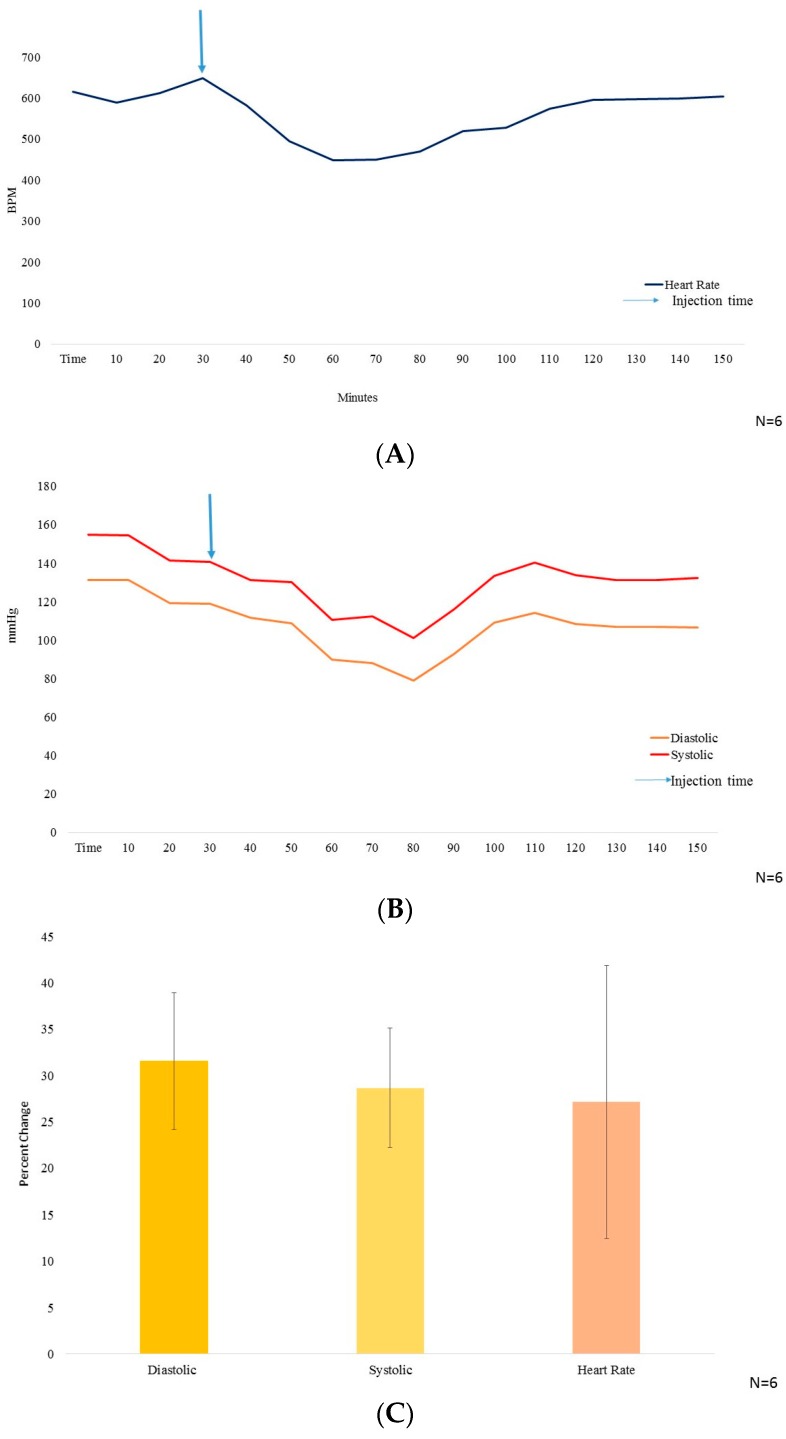
(**A**) The heart rate measurement in beats per minute (BPM) on y axis, with time (minutes) from *t* = 0–60 on x axis (N = 6); (**B**) The blood pressure (mmHg) on y axis, wherein, systolic BP is represented by red color line and diastolic BP represented by yellow color, with time from *t* = 0–160 (minutes) on x axis. 10 mg/kg BW of GA was injected i.p. at *t* = 30 min (N = 6); (**C**) Graphical representation of the % decrease in systolic and diastolic Blood pressure along with heart rate (N = 6).

The DSI small animal telemetry was implanted and the mice were allowed to recover and stabilize the blood pressure and heart rate parameters for at least 10 days post-surgery. GA at a dosage of 10 mg/kg i.p. in PBS (as vehicle) was used for measuring its effect on mice′s blood pressure and heart rate. Wild type mice with stabilized cardiac parameters were treated with GA (10 mg/kg i.p.), an acute single dose, and further monitored in the DSI telemetry system. Followed by the i.p. injection, both the blood pressure and the heart rate dropped significantly. Three to four hours post injection, the blood pressure (Figure 2A) as well as the heart rate (Figure 2B) stabilized back to normal.

The systolic and the diastolic blood pressure reduced by 28.68% ± 6.48% and 31.56% ± 7.35% and, the heart rate dropped by 27.17% ± 14.73% (Figure 2C), all peaking at 30–40 min after i.p. injection of GA.

### 2.3. In Vivo Hormonal Effect

**Figure 3:**
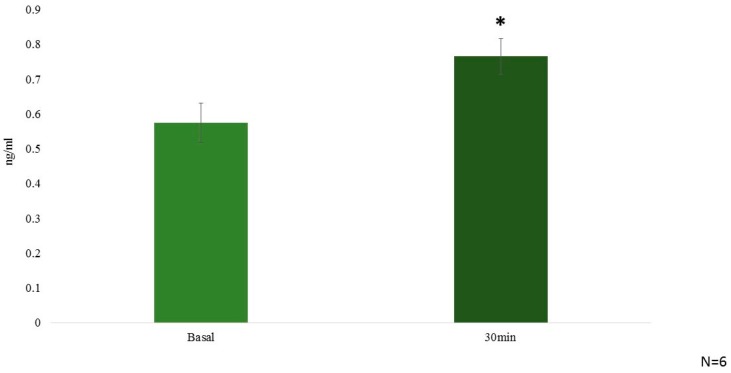
Graphical representation showing statistically significant increase in the levels of VIP (*p* value = 0.006735) upon i.p. injection of GA 10 mg/kg/bw at time zero (Basal) and after 30 min of GA stimulation (N = 6). * *p* < 0.01.

GA (10 mg/kg i.p) along with PBS as vehicle was used to treat mice. GA administration was subsequently followed by the measurement of vasoactive intestinal peptide (VIP) plasma levels. We found that i.p. injection of GA instigated a statistically significant but small rise in the VIP levels, after 30 min post i.p. injection when compared to control (Figure 3).

Furthermore, along with VIP, we observed a rise in the secretin levels in mice, peaking at *t* = 1 h [32]. Interestingly, the potential of licorice extract in mediating the release of secretin has already been previously demonstrated in dogs [33].

### 2.4. In Silico Virtual Docking

**Figure 4:**
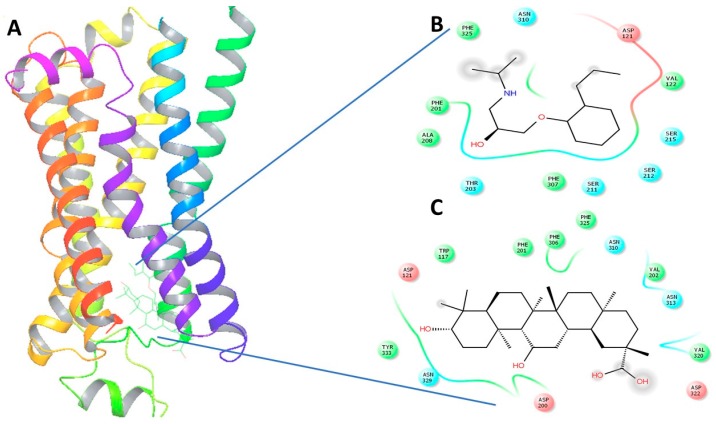
(**A**) The 3-Dimensional image of β1-AR docked with GA and alperenolol signifying same binding region for both drug molecules; (**B**) 2-Dimensional image of the interacting amino acid residues with alperenolol; (**C**) 2-Dimensional image of the interacting amino acid residues with GA molecule.

**Figure 5:**
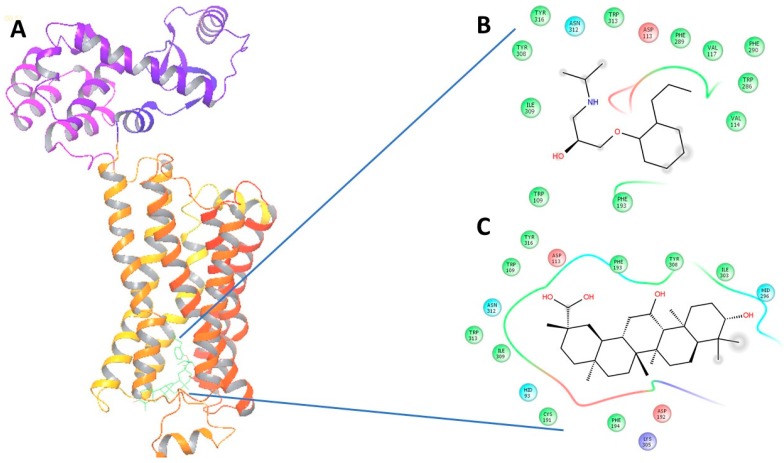
(**A**) The 3-dimensional image of β2-AR docked with GA and alperenolol signifying same binding region for both drug molecules; (**B**) 2-Dimensional image of the interacting amino acid residues with alperenolol; (**C**) 2-Dimensional image of the interacting amino acid residues with GA molecule.

Upon virtual docking between the 3D structures of GA, NE and alperenolol (retrieved from pubchem database) with β1-AR (PDB ID: 5F8U) and β2-AR (PDB ID: 3NYA), we observed the binding affinity of GA with β1-AR and β2-AR to be −56.27 and −59.51 respectively. Importantly, the binding affinity of GA was found to be higher when compared to the binding score of the control molecules, alperenolol (non-selective antagonist for all the β-AR [34]) and NE. The binding affinity of alperenolol towards β1-AR and β2-AR was observed to be −24.23 and −40.61 respectively, whereas the binding score of NE for the same was recorded to be −28.08 and −33.36 respectively (Table 1). Higher binding affinity of the GA at the same binding region (Figure 4 and Figure 5) suggest the similar mechanism of action of the two drug molecules. Additionally, it was observed that the interacting amino acid residues upon β1-AR interaction with alperenolol and GA respectively were common, such as, ASN310, ASP121, PHE201 and PHE325. Likewise, the interacting site of β2-AR with alperenolol and GA respectively, was found to be similar, with the common residues being—ILE309, TYR308, TYR316, ASN 312, TRP 313 and ASP 113.

## 3. Discussion

Adrenergic receptors are functionally involved in several important physiological functions and are implicated in various cardiovascular disorders particularly heart failure and even aging [35]. Activation of adrenergic system is well known to elevate the levels of fatty acids in plasma and result in increased gluconeogenesis by the liver. Also, the studies suggest that it mediates the inhibition of insulin release by the pancreas in order to conserve glucose [36]. There are reports signifying the relationship between physical activity and cardiovascular health and the functional role of beta adrenergic system [37]. Furthermore, age-related impairment in insulin release is documented to be significantly dependent on the beta 2 adrenergic system [38]. Along with the heart rate, β-ARs are also known to mediate vasodilation [39] and its desensitization leads to impaired β-AR vasodilation in hypertension [40].

The adrenergic stimulation by epinephrine and nor-epinephrine [6] of the heart is known to stimulate cAMP and phosphoinositide, second messenger signaling cascade [41], and therefore, in the present study we used cAMP measurement to study the activation of the adrenergic receptor by nor-epinephrine in the presence and absence of GA (10 µM). We observed a drop in the cAMP stimulation in the cells over expressing β-AR when GA was added along with NE. Separate figures for β1-AR, β2-AR and β3-AR are provided in the Supplementary Figure S1A–C). Henceforth, there is a possibility that either GA is competitively or non-competitively inhibiting NEs effect over the cells. A well-known β2-AR selective antagonist (ICI 118,551) was reported to cause a drop of 50% of NE response at 100 nM concentration and we observed a drop of 32.23% in the presence of 10 µM GA along with 100 nM NE for β2-AR. We then proceeded to perform in vivo test of the effect of GA and observed a sharp drop in the heart rate and blood pressure which were precisely measured by DSI instrument. This further suggest GAs possible role as a potential inhibitor or antagonistic molecule for β-AR. It is also very interesting to note that we observed an increase in the VIP plasma levels after the same dosage of GA. We also performed structure activity relationship study with the help of in silico analysis and used alperenolol, a well-accepted non selective antagonist for all β-AR [34]. With the help of virtual docking algorithm between crystal structures of β1-AR (PDB ID: 5F8U) and β2-AR (PDB ID: 3NYA) with alperenolol and GA, we observed the same binding location of the two molecules along with the GAs higher binding affinity in both the cases. Hence, it might be argued that both the drug molecules share the same mechanism of action. However, this remains to be proven by further experimentation. Interestingly, even though the binding energy of GA remains higher for both β1-AR and β2-AR, the in-vitro experiment shows no statistically significant drop in cAMP levels in β1-AR transfected CHO cells. Whereas, a statistically significant drop in cAMP level was observed in β2-AR transfected CHO cells, stimulated with NE, in the presence of GA. This observation might be explained because of the differences observed in the interacting amino acid residues between β1-AR with alperenolol and GA, and β2-AR with alperenolol and GA. The interacting site between β1-AR and alperenolol as well as β1-AR and GA had 4 common residues i.e., ASN310, ASP121, PHE201 and PHE325. Whereas, the interacting amino acid residues of β2-AR with alperenolol and GA had 7 common residues, which are, ILE309, TYR308, TYR316, ASN 312, TRP 313 and ASP 113, signifying a better competitive ability along with similar efficacy [42].

Since GA is capable of modulating heart rate and blood pressure after acute administration via intra peritoneal injection in vivo, hence, it might produce a similar physiological response as that of a β-AR antagonist. β-AR antagonist molecules are well recognized to be an important part of clinical practice [43] and GA, as shown in the current report, is able to reduce heart rate and blood pressure, signifying GA as a potential parent drug molecule for the same. However, a major limitation of the present study remains that the GA′s effect of reducing blood pressure and heart rate is studied in healthy wild type mice and not in the diseased mice model. Therefore, there is a need of further investigation to determine the exact effect of GA in certain pathophysiological condition. Even though, the chronic use of GA is also well known to be hypertensive [31], there is no known acute toxicological implication of GA [44]. Studies done in both rat and human shows that GA, after oral ingestion, gets rapidly hydrolyzed by commensal bacteria to aglycone. However, after injection, GA remains in its native structure with a longer half-life [19]. Therefore, in the current study, we administered GA via intra-peritoneal injection in mice and are projecting GA as a potential principal compound for the development of a future injectable drug against cardiac arrhythmia. Further studies, however, are mandatory to investigate acute effects of the drug. Since, VIP has been reported to have a vasodilatory action causing a drop in Blood Pressure (BP), therefore, it can also be estimated to aid the drop in the blood pressure in the animal model under study. The individual mice variation in the decrease of heart rate may be explained because of the GAs partial selectivity towards β2-AR, known to have variation in its expression levels [32,45]. These studies correspond to the observations reported by a parallel study indicating the potential of a β2-AR selective antagonist in 1996 by Bilman et al. [46]. Additionally, there is a study done on isolated heart (ex vivo), wherein GA was shown to activate eNOS via EE-Akt-eNOS-cGMP-PKG pathway via ERK1/2 signaling [47]. Also, studies prove that endothelial β2-AR regulate eNOS activity and consequently vascular tone, through means of PKB/AKT [48,49]. Additionally, β2-AR ligands are biased towards ERK1/2 signaling [50]. Therefore, GA as a potential antagonist against β2-AR might be a possible explanation of its ability to activate eNOS via EE-Akt-eNOS-cGMP-PKG pathway.

## 4. Experimental Section

### 4.1. In Vitro Transfected Cell and Effect of GA on cAMP Levels

The cell culture and transfection experiment was carried out as per the standardized protocol in our lab [51,52]. Briefly, minimum essential media (Invitrogen Life Technologies Limited, Hong Kong, China) augmented with 10% fetal bovine serum (Invitrogen), 100 U/mL Penicillin along with 100 µg/mL streptomycin (Invitrogen) was utilized to culture the CHO-K1 cells, obtained from American Type Culture Collections (Manassas, VA, USA). CHO cells were then incubated in a 5% (*v/v*) CO_2_ humidified chamber at a temperature of 37 °C. Cell culture was followed by the cAMP assay, wherein, a six well plate with a capacity of 35 mm/well (Costar, San Diego, CA, USA) was plated with 3 × 10^5^ cells and allowed to grow for 24 h before transfection. After 24 h of cell growth, the cells were transfected, wherein, 2 µg of the construct, receptor-pcDNA3.1, was used to transfect the CHO-K1 cells using X-treme GENE HP DNA Transfection Reagent (Roche, Mannheim, Germany), in a ratio of 1:3 (*w/v*), for each well. After two days, the transfected cells were incubated with the peptide for 45 min, which was then followed by the measurement of the intracellular levels of cAMP. The assay conducted, utilized the LANCE cAMP assay kit (Perkin Elmer, Waltham, MA, USA) with Victor x4 multilabel reader (Perkin-Elmer), which followed the manufacturer′s protocol. Also, the CHO-K1 cells transfected with pcDNA3.1 were taken as the negative control. The observed results were reported as the fold change in the intracellular cAMP levels when compared to the basal level (no agonist/antagonist control). Additionally, the cells upon transfection were used for the extraction of total RNA (TriPure reagent, Invitrogen) in order to confirm the receptor′s expression.

### 4.2. Animal Grouping (Randomization)

Male mice of age 2–3 months were used for the experiments. Only 3–4 mice were housed per cage with 12 h light and dark cycle along with free access of food (standard rodent chow) and water with temperature controlled environment at 24 °C. All the animal experiments were carried out according to the guidelines of CULATR (Committee on the use of live animals in teaching and research). The animals were acclimatized for a week before experiments and then randomized into groups based on their body weight and basal hormonal levels.

### 4.3. Serum Hormonal Level Study

Male WT mice of 2–3 months of age were used for the experiment. After randomization of mice, the mice were grouped according to their basal level of the hormones, measured using ELISA kit provided by Phoenix pharmaceuticals, INC (Phoenix Pharmaceuticals, Burlingame, CA, USA). After grouping the animals, we left them for about two weeks to acclimatize, followed by the drug administration in the animals (GA 10 mg/kg i.p. injection) and blood collection from different animals at different time points (*t* = 0, *t* = 30 min). The blood samples were procured from the tail vain of the mice and were aliquoted in different Eppendorf tubes along with heparin. Further, the serum was separated from the blood samples by centrifuging it for 15 min at 4 °C at 12,000× *g*. The hormonal levels were estimated using ELISA kit (Phoenix Pharmaceuticals, Burlingame, CA, USA) as explained above [29].

### 4.4. DSI Implantation

Mice were first sedated with the help of isoflurane (3%–4%) in 2 L of oxygen flow rate in the induction chamber for 3–5 min. The hair in the neck area were removed using depilatory cream and warm water. Betadine and 70% ethanol were used for sterilizing the area. The animal was then put on a 37 °C temperature controlled pad for operation. The dosage of isoflurane was then changed to 1%–1.5% with 2 L oxygen flow rate. For DSI implantation, approximately 1 cm cut was made longitudinally, separating the neck muscle from the connective and fat tissue, which allowed us to observe clearly the left common carotid artery. One tight suture was made near the head along with the two loose ones in the middle and another at the end of the exposed artery. Afterwards, the artery was opened by piercing with a 90 angled 27 G needle tin followed by the insertion of the pressure probe and further proceeding for 11 mm into the artery from the opening. Then the ECG positive electrode was inserted at the end of the left side of the rib cage, while the other was inserted into the right pectoral region. Further space was created till stomach and then DSI device was implanted (https://www.datasci.com/products/ implantable-telemetry/mouse-(miniature)/hd-x11). The incision was then closed using the continuous monofilament suture. The animal was administered with buprenorphine (0.3 mg/mL i.p.) for pain relief. Then until the animal recovered from anesthesia they were kept in intensive care unit for about 4–6 h. Afterwards the animals were kept back in temperature controlled 12 h day and night cycle for complete recovery for about 10 days. The blood pressure measurement and the drug administration was started after the animal got fully recovered and the diurnal blood pressure pattern returned back to normal.

### 4.5. Heart Rate and Blood Pressure Study

After DSI instalment surgery, the mice (WT *n* = 5) were allowed to recover for 10 days and then the blood pressure and heart rate change analysis were done between the days 11–15, after the base line monitoring of the same. First, the normal blood pressure (diastolic and systolic pattern) and heart rate were confirmed and then the drug treatment was done (GA 10 mg/kg i.p. in PBS). We supervised the diastolic and systolic pressure along with the heart rate for next 24 h to check the effect of the drug. PBS was given as vehicle control and the effect was analyzed after the drug responses returned back to normal.

### 4.6. Virtual Docking

The in silico virtual docking of the compounds (GA and alperenolol) was performed with their respective receptors with which they are documented to interact with, in order to computationally calculate the binding affinity towards the receptors and also to compare it to their natural ligand molecule and a known drug for the receptor (Table 1). The PDB file of the 3D model of all receptors were retrieved from RCSB protein data bank [53] (5F8U: β1-AR [54]; 3NYA: β2-AR [55]). The structural files of all the ligands used were retrieved from pubchem database (https://pubchem.ncbi.nlm.nih.gov) in SDF file format and was later converted to PDB file format using open babble software, for the docking stimulation [56]. Docking stimulation was performed using patchdock/firedock [57,58] and Schrödinger biological suite [59], and followed by the visualization of the same [60].

### 4.7. Statistical Analysis

The results were analyzed by Microsoft Excel, Fold change was calculated as treated/untreated. Significant difference was identified using student “t” test with 95% level of significance (*p* < 0.05) and was performed for in vitro and in vivo assays to compare between two groups. The graphs for in vitro analysis of cAMP results were plotted using GraphPad Prism 7.0 software and all data was expressed as mean + SEM. In vivo treatment groups of hormonal release assay were also analyzed using *t*-test and were plotted using Microsoft Excel.

## 5. Conclusions

We conclude that GA when administered via intra peritoneal injection, leads to a drop in heart rate and blood pressure in wild type mice model. This physiological effect might be due to GA′s antagonistic activity against beta adrenergic receptors and also possibly via increasing the plasma VIP levels after about 30 min of its injection. The present study, opens up a possibility for further research in GAs action on changes in heart rate and blood pressure.

## Figures and Tables

**Table 1:** Binding score after virtual docking of GA and alperenolol with 3D structures of beta adrenergic receptors (β1-AR and β2-AR).

Drug	β1-AR	β2-AR
PDB ID	5F8U	3NYA
NE	**−28.08**	**−33.36**
Alperenolol	**−38.14**	**−40.61**
GA	**−56.27**	**−59.51**

## Supplementary Materials

Supplementary materials can be accessed at: http://www.mdpi.com/1420-3049/21/10/1291/s1.
