# Supplementary material for: Glycyrrhizic Acid Reduces Heart Rate and Blood Pressure by a Dual Mechanism
Source: Molecules. 2016 Sep 27;21(10):1291. doi: 10.3390/molecules21101291 (PMC6274536; doi:10.3390/molecules21101291)
Supplement: Supplementary file 1 [file molecules-21-01291-s001.pdf]

Supplementary Materials: Glycyrrhizic Acid Reduces Heart Rate and Blood Pressure by a Dual Mechanism

Kailash Singh, Aung Moe Zaw, Revathi Sekar, Ahuja Palak, Ahmed A Allam, Jamaan Ajarem and Billy K.C. Chow

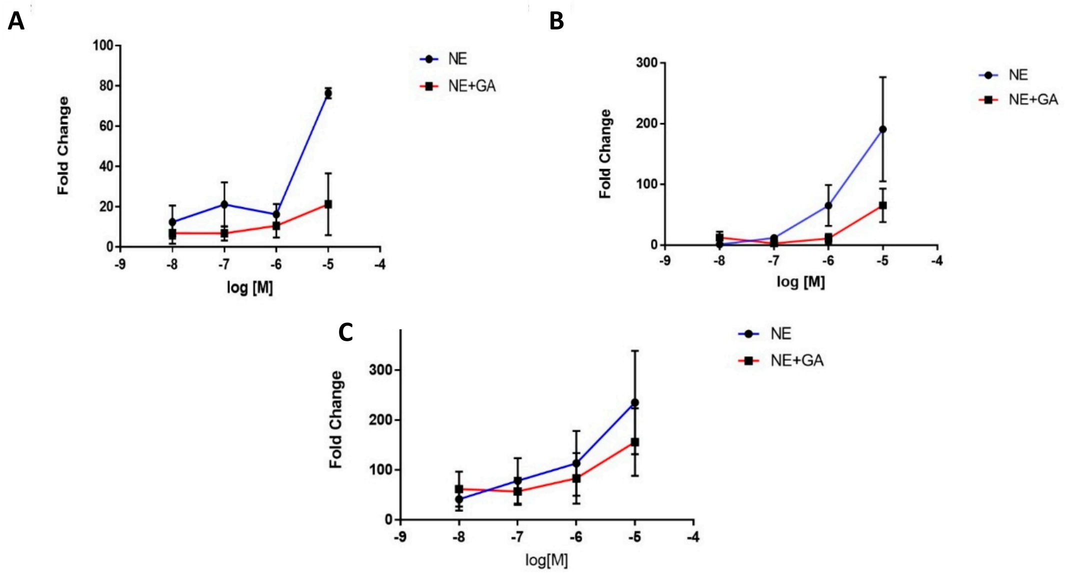

Figure S1. cAMP fold change difference at varying concentration of NE with (red line) and without (blue line) 10 μM GA on beta adrenergic receptors (**A**) beta1 adrenergic receptor; (**B**) beta2 adrenergic receptor; (**C**) beta3 adrenergic receptor.
